# Language and social-emotional and behavioural wellbeing from 4 to 7 years: a community-based study

**DOI:** 10.1007/s00787-017-1079-7

**Published:** 2017-11-15

**Authors:** Penny Levickis, Emma Sciberras, Cristina McKean, Laura Conway, Angela Pezic, Fiona K. Mensah, Edith L. Bavin, Lesley Bretherton, Patricia Eadie, Margot Prior, Sheena Reilly

**Affiliations:** 1 Newcastle University, King George VI Building, Queen Victoria Rd, Newcastle upon Tyne, NE1 7RU UK; 2 Murdoch Children’s Research Institute, Parkville, VIC Australia; 3 School of Psychology, Deakin University, Geelong, VIC Australia; 4 The Royal Children’s Hospital, Parkville, VIC Australia; 5 University of Melbourne, Melbourne, Australia; 6 La Trobe University, Melbourne, VIC Australia; 7 Griffith University, Gold Coast, QLD Australia

**Keywords:** Behavioural problems, Social-emotional problems, Language disorder, Childhood, Longitudinal study

## Abstract

Language disorder (LD) and social-emotional and behavioural (SEB) difficulties are common childhood problems that often co-occur. While there is clear evidence of these associations from clinical samples, less is known about community samples. This paper examines these associations in children aged 4–7 years from a community-based longitudinal study. 771 families provided questionnaire and assessment data at 4, 5 and 7 years. Parent-reported SEB difficulties were measured at each point (SDQ). Child language was directly assessed at 4 (CELF-P2), 5 and 7 years (CELF-4). Linear regression analysis was used to compare cross-sectional differences in mean SDQ scores between children with and without LD at each time point. Linear regression was then used to examine how patterns of language development (language disordered at three time points; never disordered; disordered at one or two time points, i.e. ‘unstable’ group) related to SEB difficulties at each age, adjusted for potential confounders, as in the previous analyses. Higher hyperactivity/inattention scores were associated with LD at each age. In fully adjusted models, there was little difference in mean emotional symptoms scores between children with and without LD. The ‘never’ LD group had lower mean SDQ scores at each time point than the ‘unstable’ group. Findings highlight that children with persistent LD from preschool to early primary school may be more likely to have concomitant SEB difficulties, particularly behavioural difficulties. Those with unstable LD may also have co-occurring SEB difficulties, showing a need for education and health professionals to monitor early language and SEB development.

## Introduction

Language disorder (LD) and social-emotional and behavioural (SEB) difficulties commonly co-occur in childhood [1]. Up to 20% of children present with LD, where standardised expressive and/or receptive language scores are > 1.25 standard deviations below the mean [2]. Approximately 6–12% of children have SEB difficulties, comprising problematic social interaction, emotional development and/or behaviour (e.g. oppositional/conduct problems, inattention/hyperactivity) [3]. Children with LD experience a diverse range of difficulties including internalising and externalising difficulties, peer relationship difficulties, and elevated rates of attention-deficit/hyperactivity disorder (ADHD) [4, 5]. Children with SEB difficulties are also at risk of LD, including difficulties with both receptive and expressive language [6, 7]. There has been growing evidence to support the links between language and behavioural difficulties, with this association demonstrated from early childhood through to adolescence [1, 8, 9].

While studies involving clinical samples have provided strong evidence of an association between LD and SEB difficulties [10, 11], less is known about these associations in community samples. In the Western Australian Pregnancy cohort (*n* = 1387), Whitehouse et al. found no evidence of an association between late-talking (low expressive vocabulary on a parent-reported checklist) at 2 years and later behavioural and emotional difficulties at any of the follow-up time points from 5 to 17 years [12]. While the late-talking toddlers were more likely than their typically developing peers to have internalising and externalising behaviour problems at 2 years, they were not found to be at any greater risk for these problems during childhood or adolescence. In contrast, a recent study using data from the Children in Focus sample (*n* = 1314) of the Avon Longitudinal Study of Parents and Children (ALSPAC) revealed that expressive vocabulary at 2 years and receptive language at 4 years made a moderate contribution to emotional and behavioural outcomes at 6 years, after adjusting for biological and social risk factors, as well as age and performance intelligence [13]. Moderate associations have been found between child vocabulary and parent ratings of behaviour problems at 3 and 5 years in a community-based sample from the Millennium Cohort Study [14]. The difference in measures used, different ways of defining language difficulties and social, emotional and behavioural problems, varying age at assessment and extent to which potential confounders have been accounted for may explain some of the mixed findings across studies.

Uncertainty remains as to whether the pattern of risk and co-occurrence of LD and SEB difficulties persists over time because few longitudinal studies have considered the nature of change in the association between SEBD and LD across multiple time points, nor have the patterns of LD and SEB difficulties been systematically mapped [5, 15]. It also remains unclear whether the association between LD and specific SEB domains change over time. In their study examining the trajectories of SEB difficulties in children with a history of LD (*n* = 103), St Clair et al. found that behavioural difficulties (hyperactivity and conduct problems), as well as emotional problems decreased between 7 and 16 years, while peer problems increased over this time [16]. Similarly, in a longitudinal study examining the prevalence and stability of SEB difficulties from 8 to 17 years in a sample of 65 students with a history of specific language impairment, different SEB domains showed different prevalence rates and different pathways over time [17].

It is evident that in order to gain a better understanding of the associations between LD and SEB difficulties, it is important to examine the different SEB domains [11, 17]. Little is known about these associations in community samples, especially the associations between LD and specific SEB domains from preschool through to the early primary school years [8]. Better understanding of the nature of these associations and the extent to which they vary over time for differing SEB domains could have implications for the design and timing of preventative interventions for both language and SEB difficulties, as well as providing insights regarding the mechanisms underpinning their relationship. To address these gaps, we utilised a community-based cohort to investigate:

1. The cross-sectional associations between LD and specific SEB domains at 4, 5 and 7 years; and
2. The nature of the associations between patterns of LD over time (categorised as language disordered at 3 time points; disordered at 1 or 2 time points; never disordered) and specific SEB domains at each time point.

## Methods

### Population

The Early Language in Victoria Study (ELVS) is an Australian prospective, longitudinal cohort study of language development from infancy to adolescence. The sampling and recruitment of its 1910 participants is extensively described elsewhere [2, 18, 19].

Briefly, children were recruited from six of metropolitan Melbourne’s 31 Local Government Areas (LGA), selected to represent geographic and socio-economic spread using the census-based Socio-Economic Index of Areas (SEIFA) [20]. Between September 2003 and April 2004 families attending their child’s 8-month Maternal and Child Health check-up in the targeted LGAs were invited into the study. Families attending an 8-month hearing screen at this time were also invited into the study, as well as a minority of families attracted by publicity in local newspapers. Exclusion criteria were children with serious disabilities or developmental delays (e.g. Down Syndrome), as well as parents with insufficient English to complete written questionnaires without an interpreter. Ethics approval was received from The Royal Children’s Hospital Human Research and Ethics Committee and from the La Trobe University Human Ethics Committee. All participating families provided written informed consent.

Parents completed a baseline postal questionnaire between the ages 7.5–10 months, and then annually around their child’s birthday between 1 and 7 years. At ages 4, 5 and 7 years, the children completed face-to-face assessments from a trained research assistant using a battery of standardised measures of speech, language and non-verbal intelligence.

### Measures

#### Social, emotional and behavioural difficulties

Parents completed the Strengths and Difficulties Questionnaire (SDQ) [21], a validated screening tool assessing five domains: hyperactivity/inattention, conduct problems, peer relationship problems, emotional symptoms, and prosocial behaviour (measured as a strength). The first four domains are summed to give a summary Total Difficulties score (possible range 0–40). Reliability (internal consistency) was conducted for the current sample. Cronbach’s alpha for each SDQ subscale across the three waves ranged from: 0.76 to 0.80 for hyperactivity/inattention; 0.38–0.65 for conduct problems; 0.42–0.60 for peer relationship problems; 0.53–0.67 for emotional symptoms; and 0.53–0.72 for prosocial behaviour. These alphas are very similar to those reported by Hawes and Dadds [22], who used a population-based sample to examine the Australian psychometric properties of the strengths and difficulties questionnaire in children from 4 to 9 years of age. In particular at age 5 and age 7, both studies demonstrate that internal consistency was strongest for hyperactivity/inattention and weakest for peer problems.

#### Language outcome measures

At 4 years, children’s language was assessed using the Clinical Evaluation of Language Fundamentals, Preschool 2nd Edition (CELF-P2), Australian and NZ Standardised Edition [23] and at 5 and 7 years the Clinical Evaluation of Language Fundamentals, 4th Edition (CELF-4) Australian Standardised edition [24] was administered. Both the CELF-P2 and the CELF-4 yield raw and standardised scores (mean of 100, SD of 15) for core, expressive and receptive language. Children scoring more than 1.25 standard deviations below the mean on their receptive or expressive language score (that is, 81 or less) were categorised as language disordered. Both the CELF-P2 and CELF-4 are widely used measures with validity demonstrated using the standardisation samples. Internal consistency for the CELF-4 using Cronbach’s alpha ranges from 0.69 to 0.91 for subtests and from 0.87 to 0.95 for receptive and expressive composite scores. The CELF-P2 internal consistency using Cronbach’s alpha ranges from 0.73 to 0.96.

#### Potential confounders

A number of confounding variables known to be associated with language development, as well as social, emotional and behavioural outcomes, were selected a priori [25]. Potential variables collected at baseline (child age 8 months) included child gender, SEIFA (census-based Socio-Economic Indexes For Areas) disadvantage score, mother’s education level, maternal age, main language spoken at home and family history of communication problems. Child non-verbal IQ, was measured during direct assessment at 4 and 7 using the Kaufman Brief Intelligence Test 2nd Edition (KBIT-2) Matrices subtest [26] and the Performance IQ subscale (Matrix Reasoning and Block Design) of the Wechsler Abbreviated Scale of Intelligence (WASI) [27], respectively. Maternal mental health score was collected at each age using the Kessler Nonspecific Psychological Distress Scale [28]. Those scoring 4 or above out of 24 were classified as having a likely mental health problem.

### Analyses

Sample characteristics by LD status were summarised at 4, 5 and 7 years using descriptive statistics. Cross-sectional differences in mean SDQ scores were compared using linear regression in unadjusted analyses and analyses adjusted for potential confounding variables as listed above. A number of linear regression models were conducted to gradually add potential cofounders to the models. The first adjusted model included socio-demographic and environmental factors: sex, mother’s education level at baseline and maternal mental health score at each age. In addition to these variables the second adjusted model included child specific and genetic factors: non-verbal IQ at 4 or 7 years and a family history of communication problems. The first but not the second adjusted model is presented in the results as the partially adjusted model, because there was little difference between the two models. The final model was the fully adjusted model including all potential confounding variables. *R* ^2^ and partial (adjusted) *R* ^2^ (correlation squared) were calculated to explore the amount of variation in language outcomes explained by each of the models overall and by each covariate. Language disorder status at each age was used to categorise children into three groups: ‘never disordered’ (no disorder at any of the three time points), ‘unstable’ (disorder at one or two time points) and ‘persistent’ (disorder at all three time points). Linear regression was then used to examine how the three groups related to SEB difficulties at each age, adjusted for the same potential confounders as per the previous analyses. Analyses were conducted using Stata version 14.1 [29].

In order to account for missing data multiple imputation was conducted, restoring the sample to the complete 1910 children recruited at baseline. Multiple imputation by chained equations was used to derive a series of 50 data sets and an imputation model was then carried out including the explanatory and outcome variables considered in the analyses. The analysis of data with multiple imputation resulted in a modest increase in the strength of associations between language and SEB variables compared to the analysis of the complete data only. Therefore, we present the analyses using the complete data only to avoid any overstatement of findings based on putting too much confidence in the imputed data.

## Results

### Sample characteristics

**Fig. 1:**
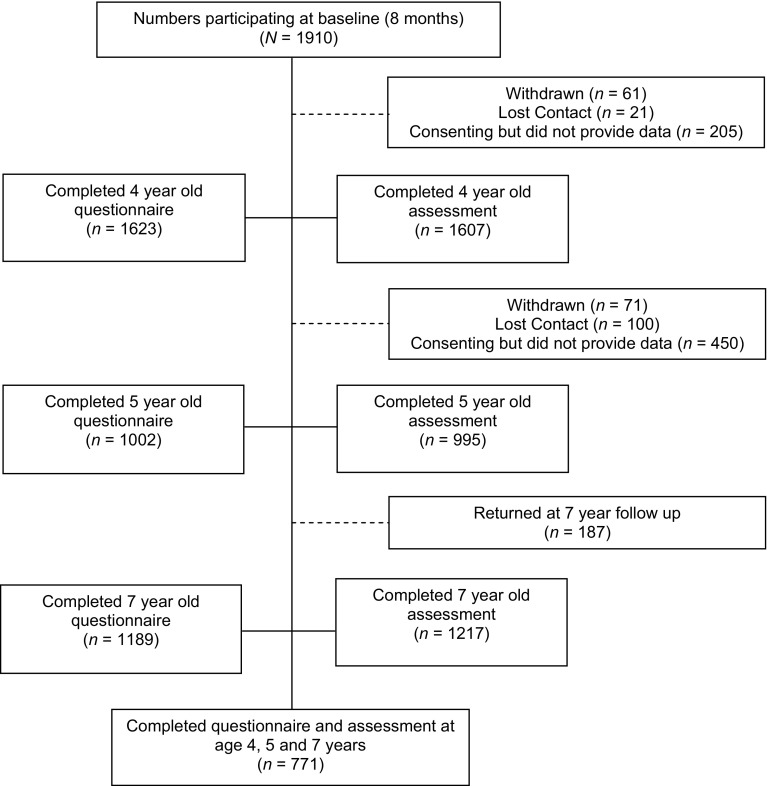
Flow chart of study participants

Figure 1 summarises the flow of participants. Of the 1910 participants recruited at baseline, 771 (40%) provided SDQ and language assessment data at all 3 waves (the in-scope sample). Table 1 shows the sample characteristics of those who provided data at all three time points (and thus were included in the analyses) compared to those who did not. Of those included, 48.6% (375/771) were males and 51.4% (396/771) were females. Compared to those not included (*n* = 1139), children included in analyses had higher mean expressive and receptive language scores at each age, higher non-verbal intelligence scores at 4 and 7 years [105.9 (14.7) vs 102.8 (14.6), *p* < 0.001] and were more likely to have mothers who had completed high school. There was no difference in maternal mental health, but maternal age at baseline was slightly higher for those included compared to those not included [32.5 (4.2) vs 31.4 (4.7), *p* < 0.001].

**Table 1:** Sample demographics by sample included versus not included

	Not included *n* = 1139	Included *n* = 771	*P* value
Female sex, *n* (%)	549 (48.2)	396 (51.4)	0.18
Expressive language score at age 4, *M* (SD)	96.5 (15.2)	102.8 (14.4)	< 0.001
Receptive language score at age 4, *M* (SD)	93.7 (15.0)	99.9 (14.1)	< 0.001
Expressive language score at age 5, *M* (SD)	98.7 (13.7)	101.2 (13.9)	0.02
Receptive language score at age 5, *M* (SD)	96.1 (14.2)	100.3 (14.0)	< 0.001
Expressive language score at age 7, *M* (SD)	96.4 (13.6)	100.1 (13.4)	< 0.001
Receptive language score at age 7, *M* (SD)	91.9 (14.5)	95.6 (13.3)	< 0.001
Non-verbal intelligence at age 4, *M* (SD)	102.3 (13.8)	106.1 (12.6)	< 0.001
Non-verbal intelligence at age 7, *M* (SD)	102.8 (14.6)	105.9 (14.7)	< 0.001
English spoken at home, *n* (%)	1045 (91.8)	753 (97.7)	< 0.001
Family history of communication problems, *n* (%)			0.80
No problem	856 (75.2)	579 (75.1)	
Speech/language problems	225 (19.8)	146 (18.9)	
Stuttering problem	58 (5.1)	46 (6.0)	
Maternal mental health (K6), *M* (SD)	3.5 (3.3)	3.4 (3.1)	0.76
Maternal education level at baseline, *n* (%)			< 0.001
Did not complete high school	298 (26.3)	149 (19.3)	
Completed high school	482 (42.5)	283 (36.7)	
University degree or higher	354 (31.2)	339 (44.0)	
Maternal age at baseline, *M* (SD)	31.4 (4.7)	32.5 (4.2)	< 0.001
SEIFA disadvantage score at baseline, *M* (SD)	1029.8 (64.9)	1045.3 (52.7)	< 0.001

At 4 years, 13% of children had LD (102/771) with a similar prevalence observed at 5 (11%) and 7 years (16%) (Table 2). Less than half of those children with LD at each time point were girls (39–41%). Non-verbal intelligence scores were slightly lower for children with LD at 4 and 7 years compared to children without LD. Children with LD tended to be less advantaged and were more likely to have a family history of communication problems compared to those without LD at each time point (see Table 2). While different children made up the LD and SEB groups at each time point, there was a consistent pattern: a larger proportion of these children had mothers who did not complete high school and a lower average SEIFA disadvantage score compared with those in the typical groups.

**Table 2:** Sample demographics at 4, 5 and 7 years by language disordered group

Characteristic	Age 4		Age 5		Age 7	
	LD^b^ *n* = 102^a^	No LD *n* = 669^a^	LD^b^ *n* = 88^a^	No LD *n* = 683^a^	LD^b^ *n* = 122^a^	No LD *n* = 649^a^
Female sex, *n* (%)	40 (39.2)	356 (53.2)	35 (39.8)	361 (52.9)	50 (41.0)	346 (53.3)
Expressive language score, *M* (SD)	80.4 (9.4)	106.2 (11.8)	78.4 (11.3)	104.1 (11.2)	82.2 (13.4)	103.5 (10.4)
Receptive language score, *M* (SD)	77.0 (8.3)	103.4 (11.3)	80.4 (10.4)	102.9 (12.3)	74.7 (10.8)	99.5 (9.5)
Non-verbal intelligence^c^, *M* (SD)	95.2 (15.2)	107.8 (11.3)	Na	Na	94.8 (10.1)	107.9 (14.5)
English spoken at home, *n* (%)	94 (93.1)	663 (99.3)	84 (95.5)	673 (98.8)	Na	Na
Family history of communication problems, *n* (%)						
No problem	67 (65.7)	512 (76.5)	59 (67.1)	520 (76.1)	79 (64.8)	500 (77.0)
Speech/language problems	23 (22.6)	123 (18.4)	23 (26.1)	123 (18.0)	34 (27.9)	112 (17.3)
Stuttering problem	12 (11.8)	34 (5.1)	6 (6.8)	40 (5.9)	9 (7.4)	37 (5.7)
Maternal mental health (K6), *M* (SD)	3.8 (3.7)	3.3 (3.0)	3.7 (3.6)	3.2 (2.8)	3.3 (3.3)	3.3 (3.1)
Maternal education level at baseline, *n* (%)						
Did not complete high school	30 (29.4)	119 (17.8)	24 (27.3)	125 (18.3)	33 (27.1)	116 (17.9)
Completed high school	44 (43.1)	239 (35.7)	34 (38.6)	249 (36.5)	58 (47.5)	225 (34.7)
University degree or higher	28 (27.5)	311 (46.5)	30 (34.1)	309 (45.2)	31 (25.4)	308 (47.5)
Maternal age at baseline, *M* (SD)	32.0 (4.4)	32.6 (4.2)	32.0 (4.6)	32.6 (4.2)	32.2 (4.4)	32.6 (4.2)
SEIFA disadvantage score at baseline, *M* (SD)	1027.3 (66.6)	1048.0 (49.7)	1015.8 (66.8)	1049.1 (49.4)	1028.4 (59.2)	1048.4 (50.8)

^a^Total sample size. This may not be the sample size for the individual characteristics

^b^LD was categorised as receptive and/or expressive language standard scores > 1.25 SD below the mean; the celf-p2 was used at 4, and the CELF-4 was used at 5 and 7

^c^The KBIT was used at age 4 and the WASI was used at age 7 for non-verbal IQ

### SEBD in children with and without LD at 4, 5 and 7 years

To address the first aim, linear regression analysis was used to examine associations between each of the SEB domains and LD at each time point. Table 3 shows the unadjusted (model 0), partially adjusted (model 1) and fully adjusted (model 2) comparisons in SDQ scores for children with and without LD at 4, 5 and 7 years. Across all three models, there was evidence of greater total difficulties for children classified with LD at 4, 5 and 7 years, compared to those without LD. In the fully adjusted models, while there was evidence of an association at 4, 5 and 7, language skills only accounted for 2.9, 1.3 and 1.8% of the variance in total difficulties, respectively. Of the potential confounder variables included, maternal mental health made a significant contribution to total difficulties at each time point, accounting for 5.7, 8.2 and 7.3% of the variance. A similar pattern was observed across all three models for hyperactivity/inattention problems with differences between groups after controlling for potential confounders being largest at 4 years (*p* = 0.004, partial *R* ^2^ = 1.1%) and 7 years (*p* < 0.001, partial *R* ^2^ = 0.7%), while slightly smaller at 5 years (*p* = 0.03, partial *R* ^2^ = 1.8%). Both maternal mental health (partial *R* ^2^ = 1.7–2.7%) and child gender (partial *R* ^2^ = 1.6% to *R* ^2^ = 2.3%) made significant contributions to the overall variance explained by the fully adjusted models at each time point. In adjusted analyses differences in conduct problems for those with LD compared to those without LD were largest at 4 years (*p* = 0.001, partial *R* ^2^ = 1.6%) and 7 years (*p* = 0.04; partial *R* ^2^ = 1.1%), while smaller at 5 years (*p* = 0.09, partial *R* ^2^ = 0.4%). The factor most strongly associated with conduct problems at each time point, respectively, was maternal mental health (partial *R* ^2^ = 3.3%; partial *R* ^2^ = 4.5%; partial *R* ^2^ = 4.6%). While there was evidence of greater peer problems for children with LD at each age in the unadjusted models, after adjusting for potential confounders evidence of an association remained at 4 (*p* < 0.001, partial *R* ^2^ = 2.3%) and 5 years (*p* = 0.01, partial *R* ^2^ = 1.0%), but not at 7 years. As with the other SEB domains, maternal mental health made a significant contribution to peer problems at each age (*p* < 0.001 for all), accounting for 1.8, 3.2 and 2.7% of the variance explained by the models.

**Table 3:** Differences in SDQ scores for children with and without LD at 4, 5 and 7 years of age

SDQ subscale	LD^a^ M (SD)	Not LD^b^ M (SD)	Model 0		Model 1		Model 2		*R* ^2^ (%)^e^
			Unadjusted mean diff (95% CI)	*P* value	Adjusted mean diff (95% CI)^c^	*P* value	Adjusted mean diff (95% CI)^d^	*P* value	
4 years of age									
Emotional symptoms	1.74 (1.65)	1.37 (1.49)	0.37 (0.06, 0.69)	0.02	0.32 (0.004, 0.63)	0.047	0.28 (− 0.05, 0.62)	0.10	6.5
Conduct problems	2.16 (1.47)	1.49 (1.41)	0.67 (0.38, 0.97)	< 0.001	0.56 (0.27, 0.86)	< 0.001	0.55 (0.23, 0.86)	0.001	9.8
Hyperactivity–inattention	4.30 (2.66)	3.18 (2.14)	1.12 (0.66, 1.59)	< 0.001	0.88 (0.42, 1.34)	< 0.001	0.72 (0.23, 1.20)	0.004	10.9
Peer problems	2.06 (1.55)	1.22 (1.39)	0.84 (0.55, 1.14)	< 0.001	0.79 (0.49, 1.08)	< 0.001	0.68 (0.36, 0.99)	< 0.001	7.7
Prosocial behaviour	6.78 (1.90)	7.53 (1.76)	− 0.75 (− 1.12, − 0.37)	< 0.001	− 0.58 (− 0.96, − 0.22)	0.002	− 0.52 (− 0.92, − 0.13)	0.01	8.2
Total difficulties	10.27 (4.97)	7.26 (4.17)	3.01 (2.12, 3.91)	< 0.001	2.55 (1.68, 3.42)	< 0.001	2.22 (1.30, 3.14)	< 0.001	16.3
5 years of age									
Emotional symptoms	1.66 (1.45)	1.30 (1.43)	0.36 (0.04, 0.67)	0.03	0.37 (0.05, 0.69)	0.03	0.25 (− 0.08, 0.59)	0.14	6.9
Conduct problems	1.79 (1.61)	1.40 (1.38)	0.39 (0.08, 0.70)	0.02	0.32 (0.01, 0.64)	0.04	0.28 (− 0.05, 0.61)	0.09	9.3
Hyperactivity–inattention	3.83 (2.56)	2.92 (2.09)	0.91 (0.43, 1.39)	< 0.001	0.74 (0.27, 1.22)	0.002	0.56 (0.06, 1.06)	0.03	10.3
Peer problems	1.52 (1.60)	0.95 (1.22)	0.56 (0.28, 0.84)	< 0.001	0.51 (0.22, 0.79)	< 0.001	0.42 (0.12, 0.73)	0.01	7.0
Prosocial behaviour	7.69 (1.70)	8.00 (1.71)	− 0.30 (− 0.68, 0.08)	0.12	− 0.17 (− 0.55, 0.22)	0.40	− 0.08 (− 0.49, 0.32)	0.69	6.4
Total difficulties	8.80 (5.05)	6.57 (4.01)	2.22 (1.30, 3.14)	< 0.001	1.94 (1.04, 2.84)	< 0.001	1.52 (0.58, 2.46)	0.002	15.9
7 years of age									
Emotional symptoms	1.78 (1.84)	1.49 (1.70)	0.29 (− 0.05, 0.62)	0.09	0.30 (− 0.03, 0.64)	0.07	0.29 (− 0.07, 0.64)	0.11	6.8
Conduct problems	1.77 (1.79)	1.25 (1.39)	0.52 (0.24, 0.81)	< 0.001	0.47 (0.19, 0.75)	0.001	0.44 (0.14, 0.73)	0.004	8.6
Hyperactivity–inattention	4.01 (2.66)	2.76 (2.23)	1.25 (0.80, 1.70)	< 0.001	1.10 (0.65, 1.54)	< 0.001	0.89 (0.42, 1.37)	< 0.001	10.9
Peer problems	1.20 (1.63)	0.87 (1.23)	0.33 (0.08, 0.58)	0.01	0.25 (− 0.003, 0.50)	0.05	0.15 (− 0.12, 0.42)	0.27	6.6
Prosocial behaviour	8.12 (1.88)	8.36 (1.62)	− 0.24 (− 0.56, 0.08)	0.14	− 0.15 (− 0.47, 0.17)	0.37	− 0.26 (− 0.60, 0.08)	0.14	5.6
Total difficulties	8.75 (6.10)	6.37 (4.42)	2.39 (1.47, 3.30)	< 0.001	2.12 (1.22, 3.02)	< 0.001	1.77 (0.82, 2.72)	< 0.001	14.1

^a^At 4 years, *N* = 102; at 5 years, *N* = 88; at 7 years, *N* = 122

^b^At 4 years, *N* = 669; at 5 years, *N* = 683; at 7 years, *N* = 649. LD defined as ≥ 1.25 SD below the mean on expressive and/or receptive language

^c^Adjusted for sex, mother’s education level at baseline and maternal mental health score at each age

^d^Adjusted for sex, mother’s education level at baseline, maternal mental health score at each age, non-verbal IQ at 4 or 7 years, family history of communication problems, SEIFA disadvantage score at baseline, maternal age at baseline, and English spoken at home at baseline

^e^ *R* ^2^ is for fully adjusted model including all potential confounding variables

Although children with LD showed poorer prosocial behaviour at 4 years in both unadjusted and adjusted models (*p* < 0.001; *p* = 0.002; *p* = 0.01, partial *R* ^2^ = 1.0), there was little evidence of a difference between those children with LD and those without at 5 and 7 years across all models. Child gender accounted for the largest amount of explained variation in prosocial behaviour at 4 years (*p* < 0.001, partial *R* ^2^ = 3.8%). While there was evidence of an association between LD and emotional symptoms in the unadjusted models at 4 and 5 years, these associations attenuated after adjusting for child gender, maternal mental health and a family history of communication problems, and there was no longer evidence of an association after all potential confounders were added to the model. The factor most strongly associated with emotional symptoms in the fully adjusted model at each age was maternal mental health (*p* < 0.001, partial *R* ^2=^3.5%; *p* < 0.001, partial *R* ^2^ = 4.4%; *p* < 0.001, partial *R* ^2^ = 5.1%, respectively).

### Comparison of SEB scores for different patterns of LD from 4 to 7 years

Linear regression was also used to investigate the nature of the associations between patterns of LD over time and SEB domains at each time point (aim 2). Table 4 shows the mean SEB scores between children classified as ‘never language disordered’ (no disorder at any of the three time points), ‘unstable’ (disorder at one or two time points) and ‘persistent’ (disorder at all three time points) from 4 to 7 years. In unadjusted analyses, there was evidence of a difference between the never LD group and the unstable and persistent pathway groups for each mean SDQ subscale score as well as the total difficulties score at 4 years. At 5 and 7 years in unadjusted analyses, differences between the LD never group and the unstable and persistent groups were evident for conduct problems, hyperactivity/inattention, peer problems and total difficulties. At each age in the adjusted analyses, the mean differences followed a pattern whereby the mean scores for conduct problems, hyperactivity/inattention, peer problems and total SDQ scores for those children in the never LD group are lower than those of the unstable LD group, and the mean scores for those children in the unstable LD group are lower than those for the persistent LD group.

**Table 4:** Comparison of patterns of LD (language disorder) by SEB (social, emotional and behavioural) difficulties scores from 4 to 7 years of age with (1) comparing unstable LD vs never LD and (2) comparing persistent LD vs never LD (*N* = 771)

	Never LD Mean (sd), *n* = 593	Unstable LD Mean (sd), *n* = 134	Persistent LD Mean (sd), *n* = 44	Unadjusted mean diff (95% CI) (1)	*P* value	Unadjusted mean diff (95% CI) (2)	*P* value	Adjusted mean diff (95% CI) (1)^a^	*P* value	Adjusted mean diff (95% CI) (2)^a^	*P* value
4 years of age											
Emotional symptoms	1.32 (1.47)	1.76 (1.68)	1.75 (1.37)	0.44 (0.16, 0.72)	0.002	0.43 (− 0.03, 0.90)	0.07	0.37 (0.09, 0.66)	0.01	0.39 (− 0.10, 0.87)	0.1
Conduct problems	1.45 (1.38)	1.90 (1.50)	2.36 (1.61)	0.45 (0.19, 0.72)	0.001	0.91 (0.48, 1.34)	< 0.001	0.36 (0.10, 0.63)	0.007	0.71 (0.25, 1.16)	0.002
Hyperactivity/inattention	3.10 (2.10)	3.75 (2.38)	5.14 (2.71)	0.66 (0.24, 1.07)	0.002	2.04 (1.37, 2.71)	< 0.001	0.42 (0.01, 0.84)	0.04	1.54 (0.84, 2.24)	< 0.001
Peer problems	1.17 (1.35)	1.78 (1.56)	2.07 (1.70)	0.60 (0.34, 0.87)	< 0.001	0.89 (0.46, 1.33)	< 0.001	0.53 (0.26, 0.80)	< 0.001	0.71 (0.25, 1.17)	0.003
Prosocial behaviour	7.53 (1.79)	7.13 (1.76)	6.90 (1.84)	− 0.40 (− 0.73, − 0.06)	0.02	− 0.63 (− 1.18, − 0.09)	0.02	− 0.27 (− 0.60, 0.07)	0.1	− 0.26 (− 0.83, 0.32)	0.4
Total difficulties	7.04 (4.06)	9.19 (4.75)	11.31 (4.89)	2.16 (1.36, 2.95)	< 0.001	4.28 (2.98, 5.58)	< 0.001	1.69 (0.91, 2.47	< 0.001	3.34 (2.01, 4.67)	< 0.001
5 years of age											
Emotional symptoms	1.29 (1.41)	1.48 (1.52)	1.68 (1.51)	0.19 (− 0.08, 0.46)	0.2	0.39 (− 0.05, 0.83)	0.08	0.13 (− 0.14, 0.40)	0.4	0.27 (− 0.20, 0.74)	0.3
Conduct problems	1.34 (1.36)	1.67 (1.45)	2.07 (1.81)	0.32 (0.06, 0.59)	0.02	0.73 (0.30, 1.16)	0.001	0.23 (− 0.03, 0.50)	0.08	0.63 (0.17, 1.09)	0.007
Hyperactivity/inattention	2.83 (2.05)	3.43 (2.31)	4.43 (2.60)	0.60 (0.20, 1.00)	0.004	1.60 (0.95, 2.26)	< 0.001	0.38 (− 0.02, 0.78)	0.06	1.13 (0.43, 1.82)	0.002
Peer problems	0.93 (1.23)	1.20 (1.31)	1.66 (1.61)	0.28 (0.04, 0.51)	0.02	0.74 (0.35, 1.12)	< 0.001	0.20 (− 0.05, 0.44)	0.1	0.53 (0.11, 0.95)	0.01
Prosocial behaviour	8.03 (1.70)	7.84 (1.68)	7.39 (1.85)	− 0.20 (− 0.52, 0.12)	0.2	− 0.65 (− 1.17, − 0.12)	0.02	− 0.09 (− 0.42, 0.23)	0.6	− 0.30 (− 0.86, 0.27)	0.3
Total difficulties	6.39 (3.96)	7.78 (4.15)	9.85 (5.61)	1.39 (0.62, 2.16)	< 0.001	3.46 (2.20, 4.72)	< 0.001	0.94 (0.19, 1.69)	0.02	2.55 (1.25, 3.86)	< 0.001
7 years of age											
Emotional symptoms	1.50 (1.72)	1.68 (1.83)	1.61 (1.51)	0.18 (− 0.14, 0.50)	0.3	0.11 (− 0.42, 0.64)	0.42	0.09 (− 0.24, 0.42)	0.6	0.16 (− 0.42, 0.74)	0.6
Conduct problems	1.24 (1.39)	1.55 (1.64)	1.93 (1.80)	0.32 (0.04, 0.59)	0.02	0.70 (0.25, 1.14)	0.002	0.20 (− 0.08, 0.48)	0.2	0.64 (0.15, 1.12)	0.01
Hyperactivity/inattention	2.70 (2.16)	3.64 (2.71)	4.30 (2.75)	0.94 (0.51, 1.37)	< 0.001	1.59 (0.89, 2.30)	< 0.001	0.77 (0.33, 1.20)	0.001	1.06 (0.29, 1.83)	0.007
Peer problems	0.82 (1.18)	1.22 (1.67)	1.39 (1.45)	0.40 (0.16, 0.64)	0.001	0.57 (0.17, 0.69)	0.005	0.27 (0.02, 0.51)	0.03	0.41 (− 0.03, 0.84)	0.07
Prosocial behaviour	8.38 (1.63)	8.25 (1.70)	7.82 (1.96)	− 0.13 (− 0.45, 0.18)	0.40	− 0.56 (− 1.07, − 0.05)	0.03	− 0.08 (− 0.39, 0.24)	0.6	− 0.71 (− 1.26, − 0.15)	0.01
Total difficulties	6.26 (4.31)	8.09 (5.92)	9.23 (5.68)	1.84 (0.95, 2.72)	< 0.001	2.97 (1.53, 4.42)	< 0.001	1.33 (0.45, 2.20)	0.003	2.26 (0.72, 3.80)	0.004

^a^Adjusted for sex, SEIFA disadvantage score at baseline, mother’s education level at baseline, maternal mental health score at each age, maternal age at baseline, non-verbal IQ at age 4 or 7, English spoken at home at baseline and family history of communication problems

There was little evidence of a difference between the never LD group and unstable and persistent groups in mean prosocial behaviour scores at ages 4 and 5 years in adjusted analyses. However, at 7 years there was evidence of a difference between the never LD group and persistent group in mean prosocial score. At 5 and 7 years, there was no evidence of a difference between the groups on mean emotional symptoms. There was a large amount of consistency between the unadjusted and adjusted analyses, with only slight attenuation across each of the models.

## Discussion

We examined the association between LD and SEB difficulties in a community sample of children assessed at 4, 5 and 7 years. We found that children with LD had greater total difficulties at each time point compared to those without LD. However, when examining the SDQ subscales, associations were evident at all three time points only for hyperactivity/inattention and conduct problems. Children with LD had greater scores on peer problems than children without LD at 4 and 5 years, but this association was no longer evident at 7 years. This finding is surprising given that previous studies have suggested children with LD are likely to experience increased peer problems from childhood to adolescence [16, 30]. It may be that those children with LD in our sample have less severe language problems and formal schooling may actually support their development of negotiation strategies and peer relationships. Those children with mild language problems who are more withdrawn may have few conversations with teachers in the preschool classroom, and may be left to play independently if there is a lot of demand on the teacher’s attention [30]. However, in the school environment, they may be encouraged to be more involved in activities and interactions that would promote their language skills and enhance positive peer relationships.

In line with findings from Hartas’ community-based study [14], there was little evidence of an association between LD and prosocial behaviour at 5 or 7 years of age. This is in contrast to recent findings from Girard et al. [8] who found in their population-based sample of 14,004 children from the Millennium Cohort Study that better expressive language at 3 years of age was associated with increased prosocial behaviour at 5 years of age. Interestingly, LD was not found to be associated with emotional symptoms at 4, 5 or 7 years in this sample. Previous studies have reported children with language difficulties are at an increased risk of emotional difficulties [9]. In the current study, maternal mental health was a more powerful explanatory factor of emotional difficulties, which may account for the lack of any associations evident between LD and emotional symptoms once maternal mental health was included in the models. Many previous studies (e.g. St Clair et al. [16]) examining the association between SEB difficulties and LD have failed to adjust for child (e.g. non-verbal intelligence) and family factors (e.g. socio-economic status) that may play a role in both language and SEB development. Findings from the current study also showed that the association between LD and hyperactivity/inattention, as well as conduct problems, may be partially explained by the shared risk of maternal mental health, but with language remaining a significant factor. These findings are consistent with those from Clegg et al.’s population-based cohort study, which showed that while language development makes an important contribution to SEB difficulties, it is only one of a number of factors to consider in the association between LD and SEB difficulties [13]. Maternal mental health may indicate an increased risk of restricted maternal responsiveness and/or emotional availability during parent–child interactions, which may contribute to LD or SEB difficulties, or indeed both [31, 32]. Alternatively, as maternal mental health was measured at each time point, maternal psychological distress could in fact be heightened and in response to the child’s LD and/or SEB difficulties [33].

These findings reflect results from previous studies suggesting that while overall SEB difficulties may be evident for children with LD, associations exist for specific SEB domains and not for others, so examining specific domains of SEB difficulties is critical [11, 17]. In addition, this study demonstrates that even after accounting for confounding variables, children with both persistent and unstable LD from 4 to 7 years have higher scores for hyperactivity/inattention and conduct problems when compared to children without LD across the preschool to early school years. This study also demonstrates that children with persistent LD consistently have higher scores of hyperactivity/inattention and conduct problems compared to children with unstable LD or no LD from 4 to 7 years. In addition, this study showed at 7 years that children with no LD were more prosocial than children with persistent LD, which may suggest that children with persistent LD are likely to have poorer prosocial skills in the early school years [34]. However, given this is an isolated finding these results need to be replicated. Interestingly, while previous studies have found an association between emotional difficulties and LD [9], there was no evidence of an association in this community-based sample. It may be that emotional difficulties are most evident in the most severe/clinical LD cases, less represented in community samples, or it might be that at a community level emotional difficulties, along with social difficulties are more evident at either an earlier or later age [12, 16].

Strengths of the study include the use of validated direct assessments of language and a validated widely used screening tool for SEB difficulties at multiple time-points across the preschool and early school years rather than using LD at an earlier time-point as the predictor of SEB difficulties at a later time-point [13, 15]. In addition, a broad range of child and family factors were adjusted for in analyses, whereas previous studies have failed to take such factors into account.

As with all longitudinal studies there were limitations. Due to higher levels of attrition and difficulties in recruiting children from more socially disadvantaged backgrounds study findings are not generalisable to the broader Australian population. In addition, a number of participants were excluded from the analyses because they did not provide language and SEB data at each age and these children had, on average, poorer language skills than those who were included. Therefore, those with more severe language difficulties and from more socially disadvantaged backgrounds may not be well represented. However, this is one of the few community samples to provide detailed language assessment at multiple time points. In addition, the differential loss of children with poorer language from the sample may have led to some of the associations found in our original analyses being understated, which is supported by the sensitivity analysis in multiple imputation we conducted in order to consider the full study cohort who participated in the initial wave of the study. Another limitation is the use of the SDQ, which was used as a measure of SEB difficulties but is a screening tool rather than a diagnostic test. However, it is widely used in epidemiological studies with acceptable levels of sensitivity and specificity.

Future research would benefit from teasing out whether long-term outcomes differ between the persistent and unstable groups. Given that in this sample average SDQ scores, specifically for the subscales of conduct problems, hyperactivity–inattention and peer problems, were greater at each time point for children in the unstable group compared to the never LD group, it is important to consider whether these children have poorer long-term outcomes, i.e. does having LD or SEB difficulties at just one or two time-points in the early years increase a child’s risk of poor outcomes in adolescence? Or should only those with persistent difficulties be of concern? We also need to understand the risk factors that contribute to those children who consistently have LD and/or SEB difficulties across their preschool and early primary school years, as well as the protective factors for those children who remain in the typically developing group across these time points despite language difficulties [35].

This study has important clinical implications. We found an association between LD and overall SEB difficulties from 4 to 7 years; the strongest association was between hyperactivity/inattention and LD. However, clinically LD and SEB difficulties may be viewed as separate entities and thus diagnostic and intervention services may not always be coordinated. Our results suggest that practitioners working with children with LD should consider the child’s SEB-adjustment, and similarly practitioners working with children with a history of SEB difficulties should consider language functioning. It is important to note that not all children with LD experience SEB difficulties and vice versa. Given the fluidity and variability in both language and SEB development throughout early childhood and into the early school years, it is important that clinicians are aware that profiles can change across time; if a child experiences a problem at one point they warrant monitoring even if they have moved into the average range on measures of language or SEB development [36].

## Conclusions

There was a clear cross-sectional association between LD and overall SEB difficulties, as well as hyperactivity/inattention at the 3 time-points spanning the preschool and early primary school years. However, these associations were not stable over time; children may move in and out of impaired and typical language and SEB categories. It does appear that at the community level, for those children with both persistent language problems and unstable language development patterns, they may be more likely to have SEB difficulties, in particular hyperactivity/inattention and conduct problems, than typically developing peers. The variability in both areas of development throughout early childhood and into the early school years highlight the importance of monitoring both a child’s language and social-emotional and behavioural development throughout childhood and considering children’s history as well as concurrent difficulties when determining levels of risk.
